# Tolerogenic Dendritic Cells in Solid Organ Transplantation: Where Do We Stand?

**DOI:** 10.3389/fimmu.2018.00274

**Published:** 2018-02-19

**Authors:** Eros Marín, Maria Cristina Cuturi, Aurélie Moreau

**Affiliations:** ^1^Centre de Recherche en Transplantation et Immunologie UMR1064, INSERM, Université de Nantes, Nantes, France; ^2^Institut de Transplantation Urologie Nephrologie (ITUN), CHU Nantes, Nantes, France

**Keywords:** autologous tolerogenic dendritic cells, transplantation, cell therapy, clinical trial, safety, mechanisms

## Abstract

Over the past century, solid organ transplantation has been improved both at a surgical and postoperative level. However, despite the improvement in efficiency, safety, and survival, we are still far from obtaining full acceptance of all kinds of allograft in the absence of concomitant treatments. Today, transplanted patients are treated with immunosuppressive drugs (IS) to minimize immunological response in order to prevent graft rejection. Nevertheless, the lack of specificity of IS leads to an increase in the risk of cancer and infections. At this point, cell therapies have been shown as a novel promising resource to minimize the use of IS in transplantation. The main strength of cell therapy is the opportunity to generate allograft-specific tolerance, promoting in this way long-term allograft survival. Among several other regulatory cell types, tolerogenic monocyte-derived dendritic cells (Tol-MoDCs) appear to be an interesting candidate for cell therapy due to their ability to perform specific antigen presentation and to polarize immune response to immunotolerance. In this review, we describe the characteristics and the mechanisms of action of both human Tol-MoDCs and rodent tolerogenic bone marrow-derived DCs (Tol-BMDCs). Furthermore, studies performed in transplantation models in rodents and non-human primates corroborate the potential of Tol-BMDCs for immunoregulation. In consequence, Tol-MoDCs have been recently evaluated in sundry clinical trials in autoimmune diseases and shown to be safe. In addition to autoimmune diseases clinical trials, Tol-MoDC is currently used in the first phase I/II clinical trials in transplantation. Translation of Tol-MoDCs to clinical application in transplantation will also be discussed in this review.

## Introduction

More than half a century has passed since the first successful renal transplantation at the Peter Bent Brigham Hospital in Boston. The procedure performed by Joseph Murray’s team showed for the first time the surgical feasibility of solid organ transplantation, at least between identical twins (1). Parallel to this achievement, research on immunosuppressive drugs (IS) demonstrated that 6-mercaptopurine (6-MP), a drug already used to treat acute lymphocytic leukemia, was able to impair immune response (2). These novel concepts of feasibility of solid organ transplantation and immunosuppressive treatment to avoid graft-versus host disease opened the doors for unrelated organ transplantation. Over the following years, advances in IS research led to the replacement of 6-MP, which is highly toxic, by cyclosporine, leading to an increase in one-year graft survival (3). Nowadays, more specific IS are being used to treat post-transplanted patients, such as mophetil mycophenolate, a B and T-cell proliferation inhibitor; tacrolimus, a B and T-cell activation inhibitor (4), and monoclonal antibodies, such as basiliximab, an IL2Rα (CD25) blocking antibody (5). However, although IS treatments favor allograft survival, these treatments are also associated with an increased risk of cancer and infections associated to the immunosuppressive state (6). Moreover, IS primarily prevents the acute rejection of allografts, whereas their efficacy in chronic rejection remains difficult to predict (7). A novel and promising strategy to minimize drugs treatment and control of chronic rejection is to combine reduced amounts of IS with immunoregulatory cell therapy in solid organ transplantation.

Cell therapy for solid organ transplantation could be performed with mesenchymal stem cells (MSC), regulatory macrophages (Mreg), tolerogenic monocyte-derived dendritic cells (Tol-MoDCs), and regulatory T (Treg) and B (Breg) cells (8). The common characteristic between these different cells is that they have been already tested in transplantation models in animals showing a benefit in terms of safety and graft survival. For example, MSC have been shown to delay heart allograft rejection (9). In humans, several clinical trials with MSC have been performed in kidney and liver transplantation (10). Among them, a large trial was carried out to compare MSC to anti-IL2Rα therapy. In this study, the authors showed a lower incidence of acute rejection and a better estimated renal function at 1 year compared to the anti-IL2Rα receiving cohort (11). On the other hand, Mreg have been shown to increase fully allogeneic allograft survival in non-immunosuppressed mice (12). Additionally, Mreg were tested in a clinical trial in living donor renal transplantation. In this study, two patients were treated with Mreg prior to transplantation followed by low doses of tacrolimus. The outcomes of this trial showed that Mreg-treated patients displayed a stable graft function after tacrolimus weaning (13). Tolerogenic bone marrow-derived DCs (Tol-BMDCs) have demonstrated to increase heart, skin, and pancreatic islet allograft survival in combination with IS (14–16). Regarding lymphoid cells, Treg therapy has been shown to be safe and effective in a pilot study in living donor liver transplantation. Indeed, 6 from 10 initial patients in this study were able to stop the immunosuppressive therapy (17). In the context of the ONE study consortium, clinical trials with Treg, Mreg, type 1 Treg cells (Tr1), and Tol-MoDCs are currently performed in living donor kidney transplantation in order to evaluate and compare the safety of these cells in transplantation (www.onestudy.org) (18). In this review, we will focus on both Tol-MoDCs and Tol-BMDCs and their translation to the clinical trial with an emphasis on their characteristics, mechanisms, and safety.

## Dendritic Cells

Dendritic cells were discovered by Steinman and Cohn back in 1973 (19, 20). However, the first clinical trial with DC therapy was carried out in 1995 in advanced melanoma patients (21). The reason to use these cells in cell therapy resides in their capacity to present antigens to T cells and to polarize the immune response; in other words, to link the innate and adaptive response (22). DCs are potent antigen presenting cells (APC), able to induce either immunity or tolerance. The first studies about the functions and characteristics of DCs demonstrated that DCs were strong stimulators of T cell response in allogeneic MLR. Additionally, the authors demonstrated the capacity of these cells to induce antigen-specific proliferation (23). Over the following years, different subsets with different ontogenies and functions have been characterized in DCs, such as conventional DC (cDC), plasmacytoid DC (pDC), Langerhans cells (LC), and inflammatory DCs. cDC commonly located in lymphoid tissues and nonlymphoid tissues are able to present antigen through major histocompatibility complex class II (MHC class II) in rodent and humans. Moreover, cDC can cross-present antigens *via* MHC class I (24). pDC, located usually in peripheral organs, are able to induce T-cell proliferation. However, pDCs are usually known to secrete high amounts of type I interferon (IFN) upon viral infection. Inflammatory DCs, also named MoDCs are derived from monocytes that infiltrate lymphoid and nonlymphoid organs as a consequence of inflammation or infection. Finally, LCs are DC skin-resident cells with the capacity to migrate to skin-draining lymph nodes. Unlike cDC, pDC, and MoDC that share the same precursor (monocyte-DC common precursor), the ontogeny of LC go back to the prenatal origin (25).

Nowadays, it has been demonstrated that the orchestration of all these DC subsets is essential for an adequate physiological response against threats, but also for the preservation of self-tolerance. In fact, it has been demonstrated that the ablation of cDC, pDC, and LCs in a model of transgenic CD11c-CRE mice, leads to a spontaneous autoimmunity (26).

### *Ex Vivo* Generated Tolerogenic DCs

**Figure 1:**
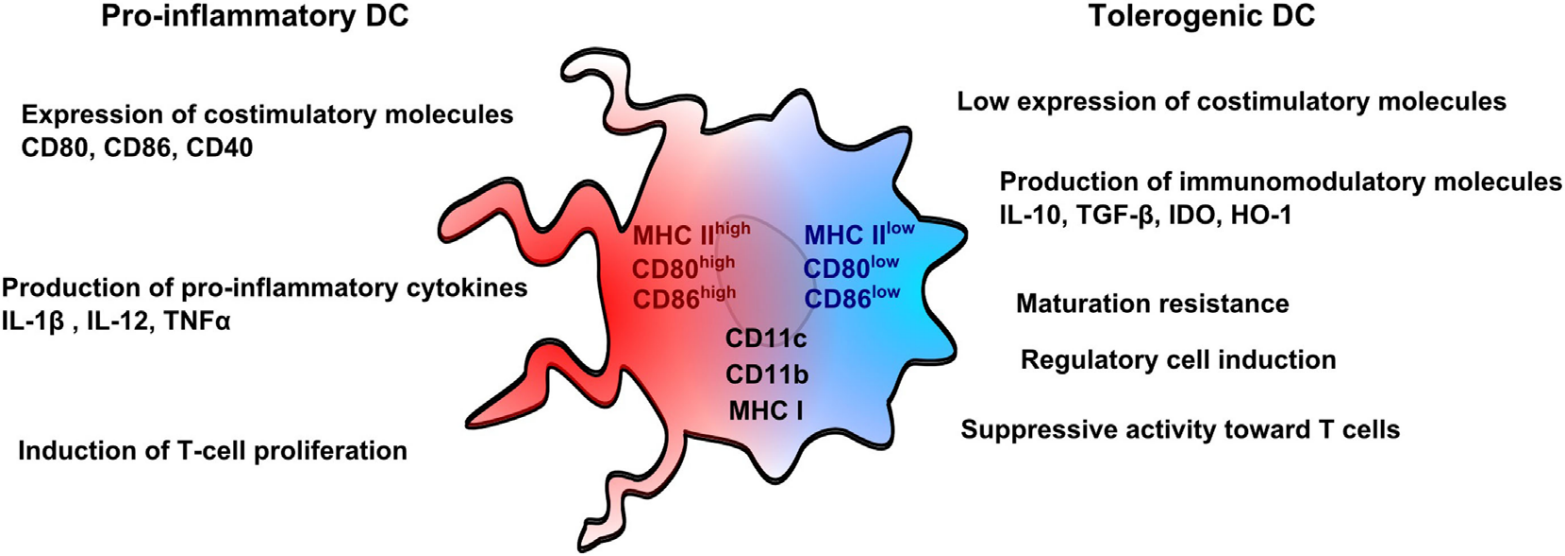
Pro-inflammatory and tolerogenic dendritic cell profile. Pro-inflammatory dendritic cells (DCs) are characterized by a high expression of costimulatory molecules (CD80 and CD86) and pro-inflammatory cytokines and by an ability to stimulate T-cell proliferation. Tolerogenic DCs display a low expression of costimulatory molecules, which are resistant to maturation, and express immunomodulatory molecules. Tolerogenic DCs have also suppressive activity toward T cells and promote regulatory T cells. Both pro-inflammatory and tolerogenic DCs express CD11b, CD11c, and MHCI.

Nowadays, rodent DCs are derived from bone marrow cells, whereas human DCs are derived from monocytes for both immunosuppressive and other therapies. Monocytes are used in humans for convenient reasons as they are more abundant than other DC precursors, and can be also manipulated *ex vivo*. From a pragmatic point of view, DCs can be differentiated *in vitro* as immunogenic or tolerogenic cells depending on the protocol. Immunogenic DCs are characterized by a high expression of costimulatory molecules, such as CD80 and CD86, a production of pro-inflammatory cytokines, such as IL1β, IL-12, and tumor necrosis factor-α (TNFα) and the ability to stimulate T-cell proliferation. In counterpart, tolerogenic DCs weakly express costimulatory molecules, are resistant to maturation, produce immunomodulatory cytokines, such as IL-10 and transforming growth factor-β (TGFβ) and impair T-cell proliferation (Figure 1). Both DCs are known to express common markers, such as CD11c, CD11b, or MHC Class I and Class II molecules (27).

As it has been previously mentioned, *in vitro* derived DC can be manipulated *ex vivo* in order to design more accurate therapies. For example, these cells can be loaded with target peptides, such as synthetic nanopeptides of MAGE-1 protein in order to direct immune response against human melanoma cells (21). On the other hand, they can be treated with inhibiting molecules associated to antigen presentation, in order to prevent pro-inflammatory response (28).

Due to this versatility and functional duality, *in vitro* derived DCs have already been used in immunogenic therapy, such as in infections (29) and cancer therapy (30), and immunosuppressive therapy, such as in allergy (31), autoimmunity (32), immunization (33), and more recently in transplantation (34).

GM-CSF is a growth factor related with bone marrow precursor mobilization and DC differentiation (35). However, the role of GM-CSF in tolerance remains unclear as its administration improves some diseases, such as myasthenia gravis, type 1 diabetes (T1D), and colitis, but its depletion improves experimental autoimmune encephalomyelitis (EAE), arthritis, nephritis, and psoriasis in rodent models (36). GM-CSF is a cytokine indispensable for *in vitro* DC generation, which is used both for immunogenic or tolerogenic DC differentiation. This dual role of GM-CSF is dichotomized by the concentration of the cytokine. Indeed, low doses of GM-CSF are associated to tolerogenic phenotypes, whereas high amounts of GM-CSF lead to immunogenic phenotypes (37).

Moreover, there is not a single standardized method to generate Tol-MoDC from monocytes in humans or Tol-BMDCs in rodents apart from GM-CSF and IL-4. Protocols to induce human and rodent tolerogenic DCs usually include several other factors, such as cytokine cocktails, organic molecules, or even clinically approved and experimental drugs (38). For example, IL-10 and TGF-β, two well-known immunomodulatory molecules, have been shown to maintain the immature phenotype of DCs (39, 40). Human Tol-MoDCs generated with IL-10 spontaneously secrete high amounts of IL-10 and are able to impair T-cell proliferation and induce Tr1 cells (41). Similarly, Tol-MoDCs generated with IL-10 and TGF-β from monocytes obtained from T1D patients was able to induce tolerance to insulin antigens. These cells express several DC markers, such as CD83, CD1a, MHC II, but not CD14 (42). Regarding small organic molecules, such as 1α,25-dihydroxyvitamin D3 (1α,25(OH)_2_D_3_ Vit D3), and prostaglandin E2 (PGE2) have been shown to induce Tol-MoDCs (38). Immature DCs treated with Vit D3 are resistant to maturation upon lipopolysaccharide (LPS) stimulation and impair allogeneic T-cell proliferation. In this study, the authors showed that Vit D3 treated MoDCs downregulated CD1a and CD14 markers (43). However, another study demonstrated that Vit D3 differentiated Tol-MoDCs express DC-SIGN (CD209), CD14, but not CD1a (44). PGE2 induces the expression of indoleamine 2,3 dioxygenase (IDO) by DC leading to a production of kynurenine that plays a role in Treg generation and allogeneic response inhibition (45). Tol-MoDCs can also be differentiated in the presence of dexamethasone (Dex) and rapamycin (Rapa). A comparative study determined that both Dex-DCs and Rapa-DCs were able to impair T-cell proliferation, but unlike Dex-DCs, Rapa-DCs displayed a mature DCs phenotype and were not able to produce IL-10 upon LPS stimulation (46). Phenotypically, it has been shown that Dex-DCs have a low expression of CD1a and CD14 and they express CD209 (44). On the other hand, it has been shown that Tol-BMDCs differentiated with Rapa are phenotypically characterized by the expression of CD11b, CD11c, CCR7, and have a low expression of MHC ClassII (47). Furthermore Dex-DCs stimulated with a cytokine cocktail (IL-6, TNFα, IL-1β, and PGE2) have been administered in patients suffering from refractory Crohn’s disease. An increase of Treg cells and a decrease of interferon-γ (IFN-γ) in blood were observed following DC injection (48). Other protocols to generate TolDCs, include genetic tools, concretely antisense oligonucleotides (AS-ODN). A study performed in nonobese diabetic (NOD)-mice showed that the injection of TolDCs modified using AS-ODN anti-CD40, CD80, and CD86 delayed diabetes onset (28).

Among these different methods, our group has adopted a protocol to generate tolerogenic DCs from mouse bone marrow cells with low doses of GM-CSF, excluding IL-4 from the classic protocol (16). This protocol, previously described by Lutz et al. (49), allowed obtaining Tol-BMDCs expressing low levels of MHCII, CD40, CD80, and CD86, and displaying resistance to maturation upon LPS stimulation. Furthermore, these Tol-BMDCs impaired allogeneic T-cell proliferation. Lutz et al. demonstrated that these cells were able to increase graft survival following a fully allogeneic vascularized heterotopic cardiac allograft, whereas we highlighted the potential of Tol-BMDCs in minor antigen skin graft survival. Alternatively, this protocol was adopted in human to generate Tol-MoDCs from blood monocytes, resulting in an equivalent profile (49). Nowadays, we are performing a first phase I/II clinical trial in kidney transplantation using Tol-MoDCs generated with low doses of GM-CSF as described previously (50). Altogether, the common phenotypical observation after tolerogenic DC differentiation showed that due to the heterogeneity of differentiation protocols it is not possible to describe a unique phenotype for these cells. However, the most common markers observed on tolerogenic DCs are CD11c and low expression of MHCII. On the other hand the expression of DC markers CD209 and CD1a, monocyte/macrophage marker CD14, and macrophage marker CD11c are variable.

## Tolerogenic DC Source

Unlike other diseases or conditions, transplantation involves the allorecognition between the two parts, the graft and the host. Allorecognition refers to an immune response against allogeneic peptides or against MHC molecules (51). The alloresponse could be differentiated depending on the nature of the interaction by direct, indirect, and semi-direct pathways. In the direct pathway, recipient T cells are activated following presentation of allogeneic MHC molecules by donor DCs and this pathway is associated with acute rejection. Indirect pathway refers to the processed allopeptides presentation by recipient DCs to autologous T cells and is usually associated to chronic rejection. On the semi-direct pathway, intact donor MHC molecules are transferred to recipient DCs through cell-to-cell contacts; the cells are then able to stimulate autologous T cells (52). Therefore, in order to avoid these types of rejection two strategies were considered: the infusion of donor-specific antigens in order to generate antigen-specific regulatory cells or in contrast, the minimization of the risk of transfer allogeneic molecules in order to avoid sensitization.

The first alternative is currently used clinically in kidney transplantation. Indeed, donor-specific transfusion (DST) is a procedure in which recipients receive a donor-specific blood transfusion in order to generate tolerance to donor antigens. A study performed in living donor kidney transplantation comparing recipients receiving DST or not, in addition to immunosuppressive therapy, showed a reduction in patients with acute rejection and an increase in patients with optimal renal function at 1 and 10 years after transplantation in the DST group (53). On the other hand, the presence of allogeneic molecules in transplantation is unavoidable and even if the efficacy of DST has been demonstrated, sensitization against HLA can occur and appears as a risk for allograft rejection (54). For this reason, the safety and efficiency of donor and recipient DCs have been discussed in DC-based therapy in transplantation.

As it has been previously mentioned, the work performed by Lutz et al. showed that Tol-BMDCs generated with low dose of GM-CSF induced an increase in allograft survival in recipient CBA mice receiving a cardiac allograft from donor B10 mice and pretreated with donor Tol-BMDCs for 7 days before the transplantation. This prolongation of allograft survival was achieved until day 100 for 70% of mice, meanwhile the mice pretreated with donor Tol-BMDCs receiving a third-party allograft from NZW mice or DC generated with GM-CSF and IL-4 increased graft survival only in 20% of mice. Moreover, in this study the authors showed that T cells cultured with allogeneic Tol-BMDCs remained unresponsive after polyclonal restimulation. These results implied that this unresponsiveness was specific (55). Another study performed by DePaz et al. in rats using donor BMDCs generated with low doses of GM-CSF showed that Tol-BMDC therapy in combination with antilymphocyte serum (ALS) was able to increase rat cardiac allograft survival in 50% of rats up to 200 days. In the same way as the previous work, the authors showed that T cells purified from transplanted mice receiving Tol-BMDCs therapy and ALS were unresponsive to donor antigens, indicating an induction of antigen-specific tolerance (56). Nevertheless, a later study using donor Tol-BMDCs or apoptotic bodies from donor Tol-BMDCs, showed that tolerance was mediated by the presentation of donor peptides (from donor cells or apoptotic bodies) by recipient DC, that inhibits CD4^+^T-cell activation and favors Treg expansion (57). Altogether these studies demonstrate the similarities of donor Tol-BMDC therapy with DST therapy. Both therapies have been shown to be partly efficient, but on the other hand, the risk of sensitization (including the development of alloantibodies) still remains. Therefore, the use of autologous tolerogenic DCs appears as a better alternative at least in terms of safety because it avoids the risk of sensitization.

In order to determine if autologous tolerogenic DCs shared a closer efficacy with donor tolerogenic DCs in transplantation, several studies have been performed. In 2005, a study performed by our team demonstrated that rat Tol-BMDCs (corresponding to the adherent fraction of rat BMDCs generated with GM-CSF and IL-4) displayed an immature phenotype were maturation resistant and were able to prolong cardiac allograft survival. Interestingly, autologous Tol-BMDCs were more efficient than donor DCs in delaying graft rejection. In this study, autologous Tol-BMDCs were injected the day before the transplantation suggesting that this time of administration was sufficient to pre-treat patients before the intervention (58). We then demonstrated that rats receiving heart allograft and treated with autologous Tol-BMDCs in combination with suboptimal doses of LF15-0195, an nuclear factor-κB (NF-κB) inhibitor, achieved definitive allograft acceptance. Moreover, we demonstrated that this tolerance was donor-specific (59). These results combined demonstrated that autologous Tol-BMDCs are even more efficient than donor Tol-BMDCs and due to its source, conceptually safer.

## Profiling Tolerogenic DC Therapy

### Combined Therapy

Previous results have shown that tolerogenic DC therapy could be improved by the addition of a complementary treatment such as ALS. However, more specific drugs have been used showing an improvement of tolerogenic DC therapy.

LF15-0195 is a NF-κB blocking agent that was previously reported to increase cardiac allograft survival in rats in short-term treatment (60). Moreover, this compound impairs the maturation of DCs (31). In combination, autologous Tol-BMDCs with a suboptimal dose of LF15-0195 induced tolerance to cardiac allograft in 92% of treated rats compared to autologous Tol-BMDCs alone, LF15-0195 alone, or rats treated with Rapa with or without autologous Tol-BMDCs. In order to determine whether this tolerance was specific, donor, recipient, and third-party skin transplantations were performed in tolerant rats. Our results showed that tolerant rats do not reject donor skin graft, but reject third-party skin graft for 16–18 days after transplantation (59). Another efficient combined therapy in transplantation was anti-CD3 antibody. Indeed, it has been demonstrated that the use of monoclonal antibody anti-CD3 leads to an increase of pancreatic islet, skin, and cardiac allograft survival in transplantation models and led to remission in T1D in autoimmune disease models (61, 62). Our results show that the combination of anti-CD3 antibody and autologous Tol-BMDCs therapy led to an increase of pancreatic islet allograft survival, associated with a decrease in CD4^+^/CD8^+^ T cell frequency, and an increase in Treg frequency. The relevance of this increased CD4^+^CD25^+^FoxP3^+^ Treg frequency and its contribution to allograft survival in this model was demonstrated by the depletion of CD25^+^ T cells with anti-CD25 antibody (15). We then confirmed the strong potential of autologous Tol-BMDCs and anti-CD3 therapy to prolong allograft survival in a model of minor antigen mismatch skin transplantation. In this model, our group found an increase in regulatory CD8^+^CD11c^+^ T cells associated with this combined therapy (16). Rapa is another drug that demonstrated an improvement of efficacy in collaboration with Tol-BMDCs in transplantation. Indeed, the injection of donor Tol-BMDCs generated with Rapa and pulsed with donor antigens followed by post-operative low doses of Rapa in heart transplantation mouse model demonstrated an increase of allograft survival. This allograft survival was related to an increase in donor-specific CD4^+^CD25^+^FoxP3^+^ Treg in the graft. To ensure the specific regulatory activity of these induced Treg, the authors performed an adoptive transfer of purified CD4^+^ T cells from treated mice to naïve mice receiving heart allograft. Adoptive Treg transfer resulted in an increase in allograft survival, indicating that tolerance was induced by this combined therapy (47). These results altogether demonstrated that autologous Tol-BMDC therapy in combination with specific drugs increased its potency.

### Administration Route and Efficacy in Non-Human Primates (NHP)

In terms of therapeutic effects, Tol-BMDCs have been shown to be efficient and safe in rodents. To ensure its safety profile for clinical trial, several works have been performed in NHP. The first study using tolerogenic DCs therapy, performed in a kidney transplantation model in NHP, showed an increase of median graft survival compared to the control group. In this study, rhesus macaques were co-treated with CTLA4-Ig and donor Tol-BMDCs generated with Vit D3 and IL-10, 7 days before the transplantation. This study demonstrated for the first time the safety and the efficiency of intravenously (IV) injected Tol-MoDCs in transplantation in NHP (63). More recently, the same authors demonstrated similar results in kidney transplantation models in NHP using autologous Tol-MoDCs pulsed with allogeneic cell membranes from donor monocytes. In this study, the authors showed an increase of graft median survival in the group treated with pulsed Tol-MoDCs compared to unpulsed Tol-MoDCs group. This improvement in allograft survival was associated with the hyporesponsiveness of T cells to donor antigens resulting in a decrease in systemic IL-17 (64). In addition, other studies have demonstrated the safety and efficacy of Tol-BMDCs in NHP notably in gene therapy. Indeed, we demonstrated the benefits of autologous Tol-BMDCs therapy to reduce immune response against a transgene product in NHP. In this study, autologous Tol-BMDCs were injected IV or intradermally (ID) in order to determine the best administration route. Our results highlighted the superiority of IV route to favor immune tolerance (65). Furthermore, several clinical trials have already been performed, confirming that ID (32, 66), intraperitoneal (48), and IV administration routes were safe and well tolerated in humans.

## Tolerogenic DC Cellular and Molecular Mechanisms

Once it has been confirmed that tolerogenic DCs improve allograft survival in rodent models and are safe in humans, the remaining question is to determine the cellular and molecular mechanisms of these cells in transplantation. To understand tolerogenic DC mechanisms (in Tol-BMDC and Tol-MoDC), it is first crucial to define the complexity of solid organ transplantation. Due to the invasiveness of the surgical procedure and the implantation of a foreign organ, even from a close source, different types of immune and non-immune cells are involved in the physiological response following transplantation. This physiological response against allograft will lead in some cases, to three expected types of rejection. The earliest one is the hyperacute rejection, in which, pre-existing recipient antidonor antibodies will react against allograft over the hours following the transplantation. This type of rejection is rare thanks to the control of HLA donor/recipient compatibility. The acute rejection is led by cellular and humoral response against allograft. This type of rejection is usually bypassed by the use of IS. Finally, the chronic rejection is led by cellular and humoral response and associated with memory cells. Chronic rejection is nowadays the main cause of rejection (67). Due to the complexity of the different types of rejection, TolDC therapy in transplantation has been evaluated on these different parameters: the migration to graft and lymphoid organs, the capacity to induce specific regulatory cells, and the ability to impair cellular and humoral response.

### Migration

It is well known that DCs have migratory skills that allow reaching different organs in order to exert different functions, depending on the maturation state. At the immature state, DCs express chemokine receptors, such as CCR2, CCR5, CCR6, CXCR4, and CXCR3 and are attracted by inflamed tissues expressing chemokines, such as CCL2, CCL5, and CCL20. At the inflammation site, DCs become mature due to the stimuli provided by the microenvironment and the antigen intake. Following their maturation, DCs overexpress CCR7 allowing them to migrate to the lymphatic system and reach the lymph nodes through CCL19 and CCL21 chemoattraction, where they present antigens to T cells. In the lymph nodes, a certain percentage of DCs will migrate to other lymphoid organs, such as spleen, thymus, and bone marrow (68). In a recent study performed in an EAE model, *in vivo* imaging of pulsed Tol-BMDCs generated with GM-CSF and VitD3 showed that these cells reached the liver and the spleen at 24 h after IV injection and remain stable for 7 days. A small amount of cells were also found in lymph nodes, thymus, and bone marrow (69). In order to support the importance of migration, DCs transduced with lentiviral vectors coding for CCR7 and IL-10 genes, prolonged cardiac allograft survival in mice, but this delay of rejection did not occur when DCs were transduced with IL-10 or CCR7 only. In this study, the authors also showed that DC transduced with CCR7 were able to migrate to LN and spleen (70). Additionally, in order to expose donor and recipient DC dynamics, a study was performed using intravital imaging in ear skin graft model in mice. In this work, authors showed that after transplantation donor dermal DCs migrate from allograft and are replaced by host DC. After donor antigen intake, these recipient DCs migrate to lymph nodes in order to present antigens to CD8^+^T cells and prime anti-allograft response. This work suggested the dynamics of DC immunotherapy *in vivo* (71).

### T Cell Inhibition

**Figure 2:**
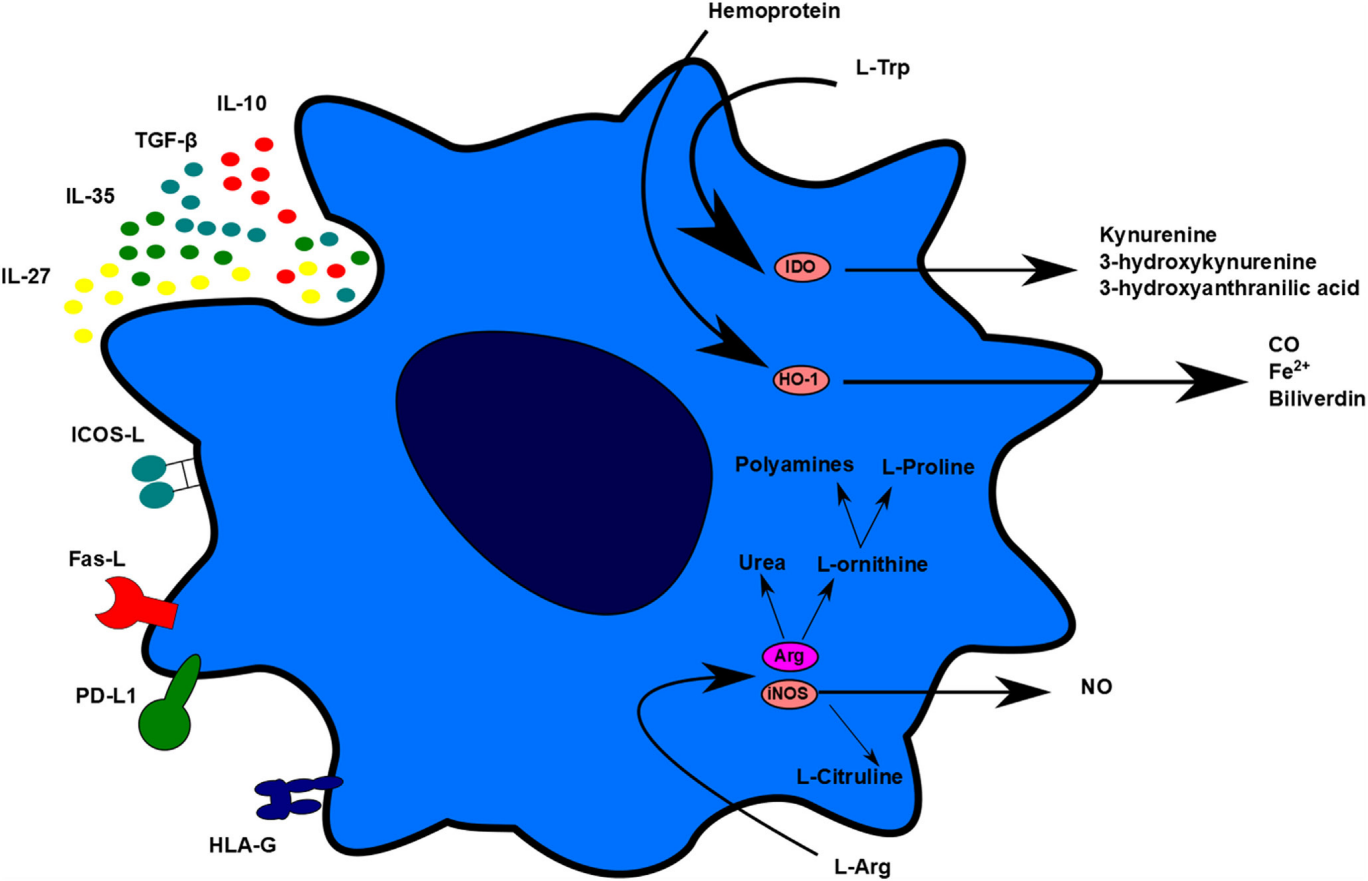
Tolerogenic dendritic cells (DC) mechanisms. Tolerogenic DCs display different mechanisms of action to impair T-cell proliferation and stimulate regulatory cell induction. These mechanisms can be differentiated in contact-dependent and contact-independent mechanisms. Contact-dependent mechanisms include Inducible T-cell costimulator-ligand, Fas, programmed death-ligand 1, and human leukocyte antigen-G. Contact-independent mechanisms include the expression of immunomodulatory cytokines and the production of small molecules, such as carbon monoxide and nitric oxide. TolDC can also act indirectly through L-Arg and L-Trp deprivation.

Even if tolerogenic DCs are able to migrate to lymphoid organs, the goal is to avoid the exacerbated proliferation of T cells in those organs and in the long term, the memory, and humoral responses. Conveniently, two common effects between the different works performed with tolerogenic DC therapy in transplantation have been observed: a decrease in the frequency of T cells in spleen, lymph nodes, and graft and an unresponsiveness of splenic T cells in contact to alloantigens (15, 58). This decrease of T-cell proliferation could be related to several tolerogenic DC molecules that lead to apoptosis, anergy, or hyporesponsivenes. There are many proposed mechanisms used by tolerogenic DCs to explain their tolerogenic activity, including contact-dependent and contact-independent mechanisms. Contact-dependent mechanisms include molecules, such as programmed-death-ligand 1 (PD-L1), Fas-Ligand (Fas-L), inducible T-cell costimulator-ligand (ICOS-L), but also other molecules, such as immunoglobulin-like transcript-2 (ILT-2), ILT-3, ILT4, HLA-G, and others. Contact-independent mechanisms could be classified into immunomodulatory cytokines, such as IL-10 and TGF-β, or enzymes that generate immunomodulatory molecules or related to nutrient deprivation, such as IDO, heme-oxygenase-1 (HO-1), inducible nitric oxide synthase (iNOS), and arginase 1 (Arg1) (Figure 2) (72).

### Contact-Dependent Mechanisms

Contact-dependent mechanisms refer to those mechanisms that need contact between lymphocyte and DCs. The inhibition of proliferation through anergy, hyporesponsiveness, or apoptosis and the differentiation of regulatory cells depend in part of the combination of surface molecules and signal integration between both cells (73). As the different types of tolerogenic DCs have different combinations of inhibitory molecules, the following description is based uniquely on contact-dependent mechanisms observed in transplantation models no matter of tolerogenic DCs type.

Inducible T-cell costimulator-ligand, expressed in immature DC, could interact with ICOS expressed by T cells in order to induce a hyporesponse which is not recovered after restimulation (74). However, a recent study in NHP in kidney transplantation using a combinatorial therapy with belatacept and ICOS-Ig human Fc fusion protein, showed no improvement of allograft survival (75).

Another well-known immunomodulatory molecule related to allograft survival and present in DCs is PD-L1. The blockade of PD-L1 accelerates skin allograft rejection in a similar way to that of anti-CTLA4 treatment (76). Similarly, the use of anti-PD-L1 antibody accelerates heterotropic cardiac allograft rejection, abrogating the effect of cytotoxic T-lymphocyte associated protein-4 (CTLA-4)-Ig (77). Moreover, a recent study showed that DCs transfected with adenovirus coding for PD-L1 was able to induce an increase of kidney allograft survival in fully mismatched rats. This improvement was associated with impairment in CD8^+^ T-cell proliferation and a decrease in pro-inflammatory cytokine production (78).

Interaction between ILT-2/ILT-4 and HLA-G in tolerogenic DC, has been shown to impair allogeneic T-cell proliferation. Nevertheless, ILT4-HLA-G pathway is more related to Treg generation (79).

Fas-ligand is another contact-dependent molecule that impairs T cell response *via* the induction of apoptosis. A study using BMDCs transfected with pBK-CMV coding for Fas-L demonstrated that these cells were able to improve cardiac allograft survival in a mouse model and to inhibit allogeneic MLR proliferation through apoptosis induction (80).

### Immunomodulatory Cytokines

Cytokines related to tolerogenic DCs, such as IL-10, TGF-β, and others have been associated with several immunomodulatory functions, such as DCs impairment of maturation, inhibition of T-cell proliferation, and regulatory cell induction.

IL-10 is a well-known immunomodulatory cytokine that has been shown to be essential for the differentiation of several regulatory populations. IL-10 activates the tyrosine kinase IL-10 receptor leading to an activation of signal transducer and activator of transcription 3. This allows an activation of the suppressor of cytokine signaling 3 that inhibits NF-κB translocation leading to a hyporesponsiveness to pro-inflammatory stimuli (81). IL-10 is expressed by tolerogenic DCs under different dynamics depending on the type of tolerogenic DCs. For example, it has been shown that MoDCs generated with IL-10 spontaneously secrete IL-10 (82). However, other Tol-MoDCs require pro-inflammatory stimulation to produce IL-10, such as Dex- and VitD3- generated TolDCs (44). IL-10 leads to a state of anergy of human CD4^+^T cells in allogeneic MLR and also after polyclonal stimulation with anti-CD3 antibody (83).

TGF-β, for its part, is a pleiotropic cytokine related to immunosuppression. In one hand, TGF-β impairs both CD4^+^ and CD8^+^ T cell differentiation, activation, and proliferation, and in the other hand, it promotes Treg expansion. In fact, it has been shown that the lack of TGF-β signaling leads to the development of autoimmune inflammatory disease due to an uncontrolled CD4^+^ activation (84). Moreover, it has been shown that Tol-BMDCs secrete TGF-β and this expression plays a crucial role in tolerance induction tolerance in several models (85). In cardiac allograft model in rat, the induction of tolerance by LF15-0195 is associated with an increase in *tgfb* expression in allograft of tolerant rats. Moreover, the adoptive transfer of splenocytes from tolerant rats to syngeneic rats receiving cardiac allograft and treated with Rapa in the presence or absence of anti-TGF-β blocking Ab showed that the tolerance was transferred and partially mediated through TGF-β (86).

Apart from classical immunomodulatory molecules, some other cytokines are potentially involved in tolerogenic DCs mechanisms. Among these cytokines, two of them share the Epstein–Barr virus-induced gene 3 (EBI3) monomer, IL-35, and IL-27. IL-35, a heterodimer of EBI3 and IL12p35, is related to immunosuppressive activity. Il-35 is mainly secreted by Treg although several studies demonstrated that APCs are also able to produce this cytokine. In fact, it has been shown that IL-35, but not other IL-12 members, is produced by Tol-BMDCs generated with Dex. In this study, the authors showed that the silencing of *Il12a* (IL-12p35) partially impaired the inhibitory effect of Tol-BMDC toward CD4^+^ T cells (87). On the other hand, IL-27 is a heterodimer composed by EBI3 and IL27p28 that acts through IL-27R (gp130 and WSX1). IL-27 impaired several pro-inflammatory functions leading to a reduced effector T-cell response, a control of neutrophil migration, and an impairment of oxidative burst (88). Nevertheless, it has been suggested a dual role for IL-27 as it displayed a suppressive role in EAE model (89), but enhanced CD8^+^ T cell anti-tumor activity in other models (90). In transplantation, IL-27 has an important relevance combined with TGF-β1. It has been demonstrated that the overexpression of IL-27 through injection of AAV-IL27 combined with Rapa improved cardiac allograft survival (86). However, monomeric function of EBI3 has been related also with tolerogenic potential in Tol-BMDCs. In fact, in heart allograft rodent model, our work highlighted that mice treated with autologous Tol-BMDCs and low dose of IS displayed an increase of splenic TCRαβ^+^CD3^−^CD4^−^NKRP1^−^DN T cells expressing high amounts of IFNγ. The increase of this double-negative regulatory population and the allograft survival were related to the EBI3-expressing autologous Tol-DCs. We showed that *in vivo* blockade of either EBI3 or IFN-γ leads to allograft rejection, demonstrating that these molecules are playing a critical immunoregulatory role in this model of allograft tolerance (14).

### Nutrient Deprivation and Other Mechanisms

On the other side, other mechanisms involving interaction between cells or nutrient competition have been observed in transplantation models for several years. These mechanisms open a new perspective on the understanding of graft microenvironment. Among these distinct mechanisms, IDO, iNOS, Arg1, and HO-1 have been related to the impairment of T-cell proliferation.

Inducible nitric oxide synthase and Arg1 are two enzymes commonly associated with macrophages. iNOS is an enzyme that metabolizes arginine and produce nitric oxide (NO) and citrulline, while arginase metabolizes arginine to ornithine and urea. Usually iNOS is known as a M1 macrophage marker and it is induced by pro-inflammatory stimuli, such as IFN-γ. The production of NO by macrophages is usually associated with pro-inflammatory response because this molecule belongs to the Reactive Nitrogen Species (RNS) family that is able to peroxidize membrane lipids in order to eliminate the inflammatory agent. On the other hand, the production of ornithine by M2 macrophages leads to the synthesis of L-Proline, which is essential for collagen production in the resolution of the inflammation (91, 92). However, it has been shown in DCs that these molecules are related to the inhibition of T-cell proliferation. To verify the implication of L-arginine in tolerance and transplantation, several studies were performed. In transplantation, a study demonstrated that the hypoproliferation of T cells isolated from grafted rats treated with Tol-BMDCs was induced by iNOS. Indeed, the use of an iNOS inhibitor (L-NMMA) allowed recovery of T-cell proliferation in treated mice (58). These results showed that iNOS was involved in allograft survival in this model. Similarly, another study demonstrated the relevance of L-arginine metabolism through iNOS and Arg1 in Tol-BMDCs. In this work, tolerogenic DCs were differentiated with retinoic acid (RA) and pulsed with OVA peptide in order to induce *in vivo* lymphoproliferation. The authors showed that Inos^−/−^ RA do not display tolerogenic potential *in vivo* in the presence of OT-II cells. This study corroborated the results observed in transplantation models (93).

Indoleamine 2,3 dioxygenase and tryptophan metabolism, have been suggested as essential factors to inhibit T, B, and NK proliferation and to induce regulatory cells. Paradoxically, it has been shown that IDO is also essential for pro-inflammatory differentiation of DCs (94). A study performed by transfecting human DCs with adenovirus coding for IDO demonstrated that these cells were able to impair T-cell proliferation. Moreover, the study showed that this effect was led by the production of several metabolites of the Kynurenine pathway including kynurenine, 3-hydroxykynurenine, 3-hydroxyanthranilic acid, but not anthranilic acid nor quinolinic acid (95). Similarly, recent findings demonstrated that IDO^+^BMDCs improved heart allograft survival in rodent models associated with an impairment of CD4^+^ response and an increase of apoptosis (96).

Heme-oxygenase-1 is an enzyme that catalyzes the conversion of Fe-Protoporphyrin-IX (Heme group) to biliverdin, ferrous ion, and carbon monoxide (CO) (97). CO is usually associated with protective anti-apoptotic effect in a large range of cells, but in lymphocytes, it is usually associated with impaired proliferation and impaired production of inflammatory cytokines (98, 99). The use of a HO-1 inductor (cobalt protoporphyrin, CoPP) or HO-1 product CO, was already tested in pancreatic islet allograft in mice. Both, the pretreatment of allograft or the pretreatment of recipient with CO or CoPP result in an improvement of allograft survival. Moreover, the delay of graft rejection was even more significant when both recipient and allograft were treated (100). Like IDO, HO-1 expression is associated to DC maturation. Indeed, HO-1 is expressed in immature DCs, but not in mature DC. Our group demonstrated that immature DCs stimulated with the HO-1 inductor CoPP preserve an immature phenotype with a low production of IL-12p70, a high expression of IL-10, and were able to impair allogeneic T-cell proliferation in humans and rats (101). Based on these results and the observation that Tol-BMDCs expressed HO-1, we then investigated the role of HO-1 in the protective effect of Tol-BMDCs in our transplantation model of heart allograft in rats. Our results highlighted that the co-treatment of grafted rats with ATDC and an HO-1 inhibitor (tin protoporphyrin IX, SnPP), impaired the beneficial effect of autologous Tol-BMDC treatment. These results suggest that HO-1 is involved in the improvement of allograft tolerance mediated by autologous Tol-BMDCs in this model (102).

Other molecules, such as thrombospondin-1 (TSP-1), PGE2, and adenosine could also influence the tolerogenic potential of tolerogenic DCs in transplantation. To test the role of these molecules in tolerance, a study was performed to compare human Tol-MoDCs differentiated with IL10, IL10/TGF-β, and IL10/IL-6. The results demonstrated that only Tol-MoDCs generated with IL10/TGF-β lost the suppressive potential *in vitro* in the presence of ARL67156 (CD39 inhibitor) or Indomethacin (PG inhibitor synthesis). However, IL-10 and TSP-1 inhibitors impaired tolerogenic potential in IL10 differentiated-DCs and IL10/IL6-DCs (103).

In conclusion, different types of tolerogenic DCs have different types of immunosuppressive mechanisms to elicit T-cell hypoproliferation.

## Regulatory Cell Induction

### Induction of CD4^+^ Treg Cells

**Figure 3:**
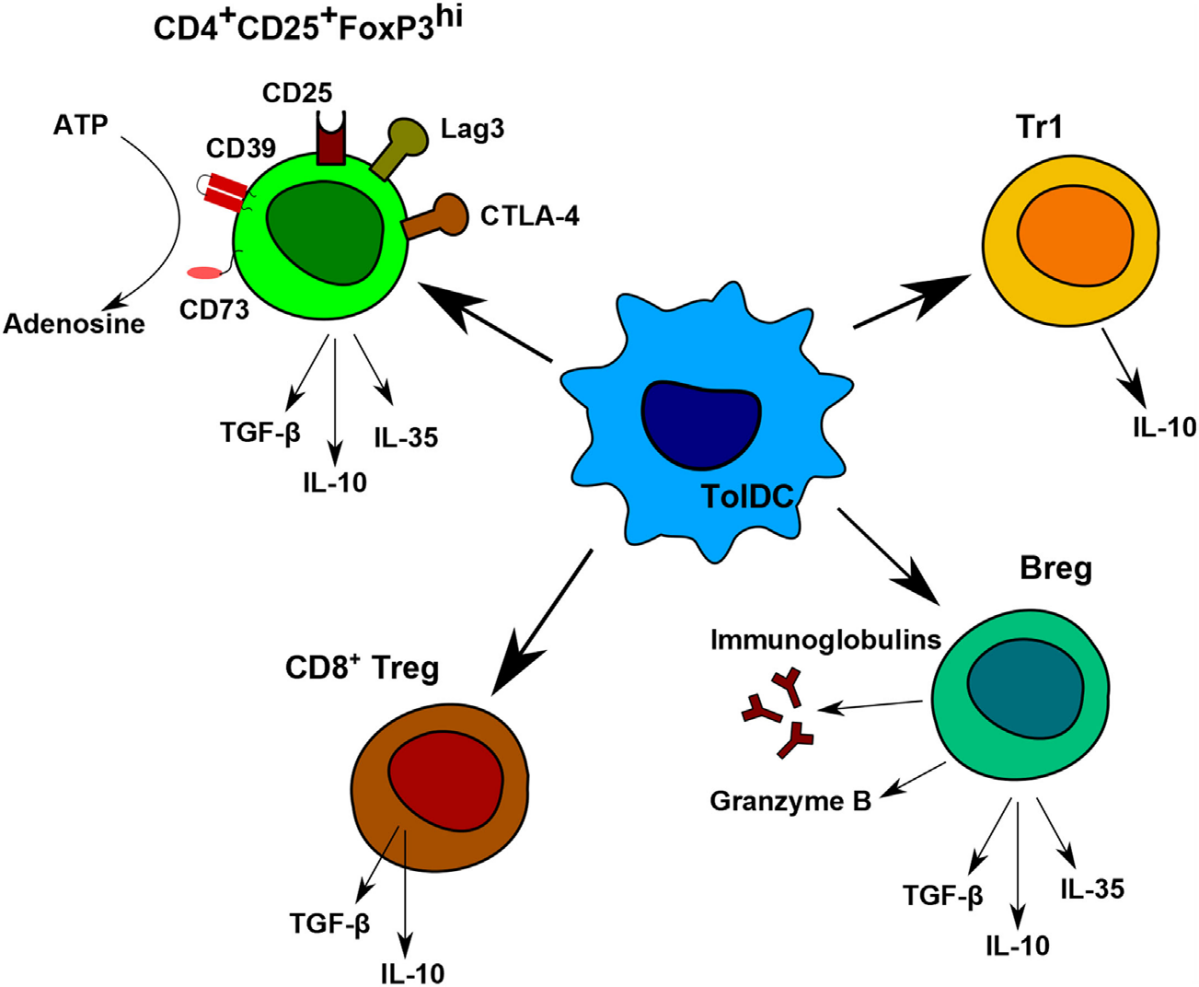
Regulatory cells induced by tolerogenic dendritic cells (DC). Tolerogenic DCs are able to induce different populations of regulatory T and B cells. Each population has different immunomodulatory mechanisms to prevent allogeneic proliferation. CD4^+^CD25^+^FoxP3^hi^ regulatory T (Treg) display contact-independent mechanisms, such as cytotoxic T-lymphocyte associated protein-4 and lymphocyte-activation gene 3 and contact-independent mechanisms by their secretion of IL-10, TGF-β, and IL-35. They are also able to produce adenosine by the degradation of ATP. Tr1 cells and CD8^+^Treg are known to produce IL-10. Breg express anti-inflammatory molecules, Granzyme B, and immunoglobulins.

Nowadays, the main goal in post-transplantation therapy is to avoid chronic rejection. To be efficient in the long term, it is essential to induce regulatory cells. Different types of regulatory cells induced or expanded by tolerogenic DCs were described in several animal models and were also observed in the first clinical trials. Among them, the main ones are Tr1 cells, induced CD4^+^CD25^+^FoxP3^hi^ Treg, CD8^+^Treg, CD3^+^CD4^−^CD8^−^Treg (104) and Breg (Figure 3) (32).

The important role of CD4^+^CD25^+^FoxP3^hi^ T cells has already been demonstrated in transplantation. Indeed, it was highlighted that the transplantation of skin allografts from tolerant mice onto new recipients, receiving donor or third-party skin allografts leads to the transfer of tolerance. In this study, the authors demonstrated that the donor allograft was not rejected while the third-party one was, meaning that tolerance was led by specific mechanisms (105). CD4^+^CD25^+^FoxP3^hi^ Treg are usually associated with several suppressive molecules, such as CTLA-4 and lymphocyte-activation gene 3 (Lag3) that trigger a signal to DCs in order to impair antigen presentation. CD4^+^CD25^+^FoxP3^hi^ Treg are also associated with the production of granzyme B and immunomodulatory molecules such as IL-10, TGF-β, and IL-35. Apart from classical contact mechanisms, CD4^+^CD25^+^FoxP3^hi^ Treg also compete with effector T cells for IL-2. The deprivation of IL-2 leads to an inhibition of proliferation and apoptosis in effector CD4 T cells (106, 107). Other mechanisms such as the production of adenosine through CD39 and CD73 have also been described (108). In transplantation models, several groups showed that tolerogenic DCs lead to the induction of CD4^+^CD25^+^FoxP3^hi^ Treg. For example, a study using Tol-BMDCs generated with Rapa have been shown to favor CD4^+^CD25^+^FoxP3^hi^ Treg population. In this study, the injection of syngeneic Rapa-DCs pulsed with donor antigens induced tolerance to heart allograft. The adoptive transfer of T cells from tolerant mice to syngeneic mice transplanted with heart allograft from the same source promote an increase in allograft survival due to the transfer of CD4^+^CD25^+^FoxP3^hi^ Treg (47). Moreover, our recent studies in pancreatic islet allograft transplantation demonstrated that CD4^+^CD25^+^FoxP3^hi^ Treg were increased in spleen, lymph nodes and graft of mice treated with autologous Tol-BMDCs and anti-CD3. As mentioned above, this Treg induction was essential for graft prolongation (15).

Other Treg-cell types commonly observed in tolerogenic DC therapy are Tr1 and Tr1-like cells (104). Tr1 are associated with a high expression of IL-10 after specific stimulation and the expression of Lag3 and CD49b markers (109). These Tr1 cells could be induced by Tol-MoDCs generated with IL-10 through the HLA-G/ILT4 pathway (41). Furthermore, it has been shown that Tol-MoDC generated with VitD3 stimulate the generation of Tr1-like cells with a high expression of IL-10 and are able to impair allogeneic T-cell proliferation (110). Interestingly, these Tr1-like cells are induced by contact with Tol-MoDCs notably by PDL-1/PD1 interaction (44). Tr1 have been shown to play an important role sustaining graft CD4^+^CD25^+^FoxP3^hi^ Treg from the spleen through the expression of IL-10 in pancreatic islet allograft (111). These results indicated a network between different tolerogenic populations in order to prolong allograft survival. Another study demonstrated that Tr1-like cells (IL-10^+^FoxP3^−^CTLA-4^+^CD25^hi^Egr2^+^ cells) could be differentiated from anergic IL-10^−^FoxP3^−^CTLA-4^+^CD25^+^Egr2^+^T cells following their interaction with immature DCs (112). These Tr1-like cells were able to inhibit T-cell proliferation *in vivo* and *in vitro* in an antigen-specific manner (112).

Another CD4 T cell regulatory population potentially associated with tolerogenic DCs are the iTR35 cells. iTR35 are regulatory cells that suppress through IL-35 production but not through IL-10 nor TGF-β. Interestingly, these cells do not express FoxP3. iTR35 are generated *in vitro* with IL-10 and IL-35 but *in vivo* they are present in models such as intestine infection and cancer (113). IL-35 is highly expressed on human Dex induced-tolerogenic DCs after pro-inflammatory stimulation with IFN-γ, CD40-L, or LPS (87). However, the role of IL-35 secreting tolerogenic DCs and iTr35 differentiation *in vivo* remains a conjecture today.

### Induction of Non-CD4^+^ Regulatory Cells

Apart from CD4 regulatory cells, there are other regulatory populations involved in TolDC therapy in transplantation such as CD8 Treg and Breg. CD8 Treg cells are less characterized than CD4^+^ regulatory cells but they are known to express IL-10 and TGF-β (114). In mice and humans, splenic CD8^+^CD122^+^PD-1^+^ population is associated to an increased allograft survival (115) and also to an anti-inflammatory and suppressive function in other models (116). Moreover, there are several works that have demonstrated a link between tolerogenic DCs and CD8 Treg induction. In humans, a study performed in 2002 showed that antigen-specific CD8 T cells with suppressive activity are generated in healthy volunteers treated with immature DCs pulsed with influenza matrix peptide (117). Another study performed in NHP showed that animals treated with CTLA4-Ig and donor Tol-BMDCs prior to kidney transplantation developed an increased proportion of donor-specific Eomesodermin^lo^CTLA4^hi^CD8^+^ T cells. This population is associated with an improvement in allograft survival (118). In our experiments, an increase of CD8^+^CD11c^+^ T cells was observed in a model of allograft skin transplantation in mice treated with autologous Tol-BMDCs and low doses of anti-CD3 antibody. The adoptive transfer of CD8^+^ T cells purified from these animals was able to prolong allograft survival in new transplanted mice. These results suggest that CD8^+^CD11c^+^ T cells induced by autologous Tol-BMDCs could be regulatory cells (16).

Although B cells are well known to promote allograft responses, there is growing evidence that in some circumstances B cells also contribute to the maintenance of transplant tolerance (119). Different populations of regulatory B cells have been described from immature state to plasma cells. Breg effects were described to be mediated by immunomodulatory cytokines such as IL-10, IL-35, and TGF-β, contact-dependent mechanisms, cytotoxic activity mediated by Granzyme B and also by immunoglobulin secretion (120). In transplantation, the ability of B cells to delay graft rejection has already been demonstrated in different rodent transplantation models (121, 122) Furthermore, studies from our team and others demonstrated that the adoptive transfers of splenic B cells from tolerant animals (either total B cells or B cell subsets) were able to delay graft rejection both in heart transplantation in rats and in a mouse model of skin transplantation (123, 124). Other reports highlighted the induction of Breg following Tol-MoDC therapy. Interestingly, in the first phase I clinical trial with Tol-MoDC therapy in type 1 diabetic patients, an increase of B220^+^CD11c^+^ population was observed in the blood of patients treated with Tol-MoDCs modified with ODN anti-CD40/CD80/CD86 during the first 6 weeks. This phenotype coincides with a regulatory population (32). Additionally, the same authors demonstrated the contribution of suppressive B cells to control the development of T1D in NOD mice after Tol-BMDC treatment. In this study, the authors suggested that the expansion of pre-existing IL-10^+^ B cells and the “*de novo*” generation from CD19^+^ B cells could be mediated by the secretion of RA-DCs from Tol-BMDCs (125). However, the link between tolerogenic DCs, regulatory B cells, and allograft tolerance remains unclear.

Altogether these results show that tolerogenic DCs are able to induce regulatory cells leading to a regulatory network that could improve the allograft acceptance.

## Where Do We Stand?

From the first DCs vaccines back in 1995 (33) until today, the expectation on DCs therapy have increased due to the safety and potential demonstrated in animal models and in humans. Nowadays, four clinical trials using Tol-MoDCs in autoimmune diseases have already been completed (32, 48, 66, 126) (Table 1). First clinical trial was performed in insulin-requiring T1D patients. In this clinical trial, seven patients received Tol-MoDCs modified with ODN anti-CD40/80/86 and three were treated with unmodified Tol-MoDCs. An increase in B220 B cells and no adverse effects were observed (32). The second clinical trial using Tol-MoDCs was performed in rheumatoid arthritis patients. In this study, 18 HLA-positive RA patients were divided into two cohorts, patients from the first one received a low dose of Tol-MoDC (one million cells) and the others received high dose (five million cells). Tol-MoDCs used in this study were modified with an NF-κB inhibitor and pulsed with four citrullinated peptides. No adverse effects were observed. Additionally, the authors observed an increase in circulating Treg cells and a decrease in IL-6 expression in T cells in response to vimentin_447–455_ Cit450 (66). The third clinical trial using Tol-MoDCs was performed in patients suffering from refractory Crohn’s disease. In this clinical trial, 12 patients were divided in 6 cohorts, receiving 2, 5, or 10 million Tol-MoDCs in a single dose or biweekly. Despite that no adverse effects were observed in most patients, three of them withdrew the study due to worsening of the disease. Additionally, the authors found an increase in Treg cells and a decrease in IFN-γ in blood (48). Finally, the most recent clinical trial using Tol-MoDCs was performed in rheumatoid and inflammatory arthritis. In this study, 13 patients were divided in four cohorts, receiving 1, 3, or 10 millions cells and three patients receiving saline solution. Tol-MoDCs used in this clinical trial were differentiated using Dex and vitD3 and loaded with autologous synovial fluid. The outcome of this study showed that the treatment was safe and feasible. Moreover hypertrophy, vascularity and synovitis were stable in all cohorts and in placebo-treated patients. Nevertheless, two patients that have received 10 millions cells showed a decrease in synovitis score (126). Apart from these studies, there are many other ongoing clinical trials focused on other pathologies, such as allergy or multiple sclerosis. Among the ongoing clinical trials using Tol-MoDCs, we supervise a phase I/II clinical trial in kidney transplantation at Nantes university hospital (NCT02252055). This trial will evaluate the safety of autologous Tol-MoDCs in patients receiving living donor kidney transplantation and a minimized immunosuppression. In this trial, autologous Tol-MoDCs are generated in the presence of low-dose GM-CSF as the only cytokine used. These Tol-MoDCs are characterized by a weak capacity to stimulate allogenic T cells and a suppression of the proliferation of stimulated T cells. Furthermore, they are resistant to maturation stimuli. Patients receive their Tol-MoDCs the day before transplantation by intravenous route at a dose of one million/kg [for review (34)]. The team of Angus Thomson also evaluate the potential of Tol-MoDC in transplantation. In this trial, patients receive donor-derived Tol-MoDCs one-week prior to liver transplantation (NTC03164265) [for review (127)]. Due to the outcomes of these clinical trials, at least in terms of safety and biological effect, Tol-MoDC therapy appears more and more as an interesting strategy to treat several diseases. However, more clinical trials must be performed in order to find out the adequate dose, injection conditions, and associated drugs to efficiently treat patients.

**Table 1:** Completed clinical trials.

Differentiation protocol	Disease	Patients	Cohorts	Biological effect/safety
Tolerogenic monocyte-derived dendritic cells (Tol-MoDC) modified with oligonucleotides (ODN) anti-CD40/80/86	Type 1 diabetes	10	Unmodified Tol-MoDC ODN Tol-MoDC	Increase in B220 B cells in blood No adverse effects

Tol-MoDC treated with nuclear factor-κB inhibitor and pulsed with citrullinated peptides	Rheumatoid arthritis	18	Low dose (1 million cells) High dose (5 millions cells)	Increase in Treg in blood Decrease in T-cell response to vimentin 447–455 Cit450 No adverse effects

Tol-MoDC differentiated with dexamethasone (Dex) and IL-6, TNF-α, IL-1β, and prostaglandin E2	Refractory Crohn’s disease	12	2, 5 or 10 millions cells Single dose or biweekly	Increase in Treg in blood Decrease in interferon-γ (IFN-γ) in blood Three patients withdrew due to disease worsening

Tol-MoDC differentiated with Dex and loaded with autologous synovial fluid	Rheumatoid and inflammatory arthritis	13	1, 3, 10 millions cells	No biological effect in blood No adverse effects

## Conclusion

Tolerogenic DCs have a solid background that corroborates their usefulness in transplantation, but also to treat autoimmunity and allergy diseases. Despite the different methods to generate them and the different models used, the common features of tolerogenic DCs converge in a low expression of costimulatory and presentation molecules, a maturation resistance, a high expression of immunomodulatory molecules, a low expression of pro-inflammatory molecules, and an impairment of T-cell proliferation. Moreover, tolerogenic DCs induce regulatory populations that are related to the protection of allograft in the long term. More importantly tolerogenic DCs have been proved to be safe supporting the feasibility of this cell therapy in humans. Finally, results confirming the efficacy and safety of autologous Tol-MoDC in humans in transplantation will be evaluated in the following years.

## Author Contributions

All authors contributed in discussing the topic and wrote the manuscript.

## Conflict of Interest Statement

The authors declare that the research was conducted in the absence of any commercial or financial relationships that could be construed as a potential conflict of interest.
